# Overrepresentation of Enterobacteriaceae and *Escherichia coli* is the major gut microbiome signature in Crohn’s disease and ulcerative colitis; a comprehensive metagenomic analysis of IBDMDB datasets

**DOI:** 10.3389/fcimb.2022.1015890

**Published:** 2022-10-04

**Authors:** Babak Khorsand, Hamid Asadzadeh Aghdaei, Ehsan Nazemalhosseini-Mojarad, Bahareh Nadalian, Banafsheh Nadalian, Hamidreza Houri

**Affiliations:** ^1^ Gastroenterology and Liver Disease Research Center, Research Institute for Gastroenterology and Liver Diseases, Shahid Beheshti University of Medical Sciences, Tehran, Iran; ^2^ Basic and Molecular Epidemiology of Gastrointestinal Disorders Research Center, Research Institute for Gastroenterology and Liver Diseases, Shahid Beheshti University of Medical Sciences, Tehran, Iran; ^3^ Foodborne and Waterborne Diseases Research Center, Research Institute for Gastroenterology and Liver Diseases, Shahid Beheshti University of Medical Sciences, Tehran, Iran

**Keywords:** inflammatory bowel disease, Crohn’s disease, ulcerative colitis, Metagenomics, gut microbiome, Proteobacteria, Enterobacteriaceae, *Escherichia coli*

## Abstract

**Objectives:**

A number of converging strands of research suggest that the intestinal Enterobacteriaceae plays a crucial role in the development and progression of inflammatory bowel disease (IBD), however, the changes in the abundance of Enterobacteriaceae species and their related metabolic pathways in Crohn’s disease (CD) and ulcerative colitis (UC) compared to healthy people are not fully explained by comprehensive comparative metagenomics analysis. In the current study, we investigated the alternations of the Enterobacterales population in the gut microbiome of patients with CD and UC compared to healthy subjects.

**Methods:**

Metagenomic datasets were selected from the Integrative Human Microbiome Project (HMP2) through the Inflammatory Bowel Disease Multi’omics Database (IBDMDB). We performed metagenome-wide association studies on fecal samples from 191 CD patients, 132 UC patients, and 125 healthy controls (HCs). We used the metagenomics dataset to study bacterial community structure, relative abundance, differentially abundant bacteria, functional analysis, and Enterobacteriaceae-related biosynthetic pathways.

**Results:**

Compared to the gut microbiome of HCs, six Enterobacteriaceae species were significantly elevated in both CD and UC patients, including *Escherichia coli*, *Klebsiella variicola*, *Klebsiella quasipneumoniae*, *Klebsiella pneumoniae*, *Proteus mirabilis*, *Citrobacter freundii*, and *Citrobacter youngae*, while *Klebsiella oxytoca*, *Morganella morganii*, and *Citrobacter amalonaticus* were uniquely differentially abundant and enriched in the CD cohort. Four species were uniquely differentially abundant and enriched in the UC cohort, including *Citrobacter portucalensis*, *Citrobacter pasteurii*, *Citrobacter werkmanii*, and *Proteus hauseri*. Our analysis also showed a dramatically increased abundance of *E. coli* in their intestinal bacterial community. Biosynthetic pathways of aerobactin siderophore, LPS, enterobacterial common antigen, nitrogen metabolism, and sulfur relay systems encoded by *E. coli* were significantly elevated in the CD samples compared to the HCs. Menaquinol biosynthetic pathways were associated with UC that belonged to *K. pneumoniae* strains.

**Conclusions:**

In conclusion, compared with healthy people, the taxonomic and functional composition of intestinal bacteria in CD and UC patients was significantly shifted to Enterobacteriaceae species, mainly *E. coli* and *Klebsiella* species.

## Introduction

Inflammatory bowel diseases (IBD) refer to chronic and severely debilitating, immune-mediated disorders that cause persistent inflammation in the gastrointestinal (GI) tract, comprising Crohn’s disease (CD) and ulcerative colitis (UC) (Loftus and Sandborn, 2002; Conway et al., 2017). Patients with IBD experience significant morbidity and poor quality of life, due to the protracted disease course frequently leading to hospitalizations and surgery (Bernstein et al., 2012). Although the etiology of IBD remains rather obscure, it is recognized to be multifactorial and both hereditary and environmental factors (e.g., gut microbiota, lifestyle, dietary factors, and heightened hygiene) contribute to disease development (Piovani et al., 2019).

Colonization and development of a stable intestinal microbiome is critical for efficient innate and adaptive mucosal immunity and continuous epithelial renewal. It has been long believed that the altered host-microbe homeostasis, defined as microbial *dysbiosis*, contributes to the pathogenesis and development of autoimmune and inflammatory disorders of the intestine, particularly IBD (Zechner, 2017). Recently, the literature suggests that some members of the gut microbiome that are better described as *pathobionts*, which are related to inflammatory-mediated disorders and depend on genetic defects or other environmental factors to cause disease, play an essential role in the development and progression of IBD (Zechner, 2017; Mirsepasi-Lauridsen et al., 2019; Chandra et al., 2021).

Much scientific evidence has confirmed an expansion in the proportion of potentially harmful Proteobacteria in IBD patients (Morgan et al., 2012). More importantly, the number of converging strands of research into IBD have reported an increase in the proportion of pathobiont strains belonging to the Enterobacteriaceae family in the gut of patients with IBD (Lupp et al., 2007; Mukhopadhya et al., 2012a; Janfaza et al., 2017; Baldelli et al., 2021). Moreover, several murine models with chemically-induced colitis have proved the notion that Enterobacteriaceae seem to have a growth advantage over other members of the intestinal microbiome in the inflamed mucosa (Håkansson et al., 2015; Munyaka et al., 2016). Furthermore, an increment in bacteria belonging to Enterobacteriaceae has been described to be associated with an aggressive disease course with a higher risk of treatment failure in CD patients (Olbjørn et al., 2019). The classic example is an epithelium-associated invasive *Escherichia coli* strain, referred to as adherent-invasive *E. coli* (AIEC) that is frequently isolated from CD patients and has been shown to adhere to and invade intestinal epithelial cells (Mukhopadhya et al., 2012b; Nadalian et al., 2021). In light of existing evidence considering the prevalence of AIEC in IBD, this pathobiont is increased in IBD patients as opposed to healthy individuals.

Recent advances in high-throughput sequencing technologies have enabled researchers to identify and characterize disease-related microbiome entities, particularly in cases of IBD (Malla et al., 2019). Analysis of the metagenomics dataset can address not only microbial community structure but also microbiome-derived metabolics and related functional pathways. Hence, these high-throughput sequencing technologies have made important contributions to studying host-microbe interactions (Zünd et al., 2021). As interactions of gut pathobiont strains of Enterobacteriaceae with enteric epithelial cells and the immune system could be relevant to the development, progression, or disease relapse in IBD, we aimed to conduct a comparative metagenomic analysis to identify the structural and functional composition of gut pathobionts belonging to Enterobacteriaceae in IBD.

## Methods

### Input data characteristics and collection procedure

In this study, the metagenomic datasets, as well as the relative metadata were selected from Integrative Human Microbiome Project (HMP2 or iHMP) publicly available on the Inflammatory Bowel Disease Multi’omics Database (IBDMDB) website (https://ibdmdb.org), which included 125 healthy people, 191 CD, and 132 UC patients. All raw shotgun metagenomics sequencing data were downloaded from NCBI Sequence Read Archive (SRA), under Bioproject accession numbers PRJNA400072 (Franzosa et al., 2019), PRJNA398089 (Lloyd-Price et al., 2019), and PRJNA389280 (Schirmer et al., 2018). The metagenomics data belonged to the patients who had undergone antibiotic and anti-inflammatory treatments medications, as potential confounding effects.

### Trimming and taxonomic profiling of metagenomic data

Original raw reads were trimmed using trimmomatic (version 0.39) (Bolger et al., 2014) to remove adapter contamination and poor-quality bases. Sliding window size 4, minimum length 50, leading 20, and trailing 20 were used as Trimmomatic parameters. Then, Bowtie2 (version 2.3.5.1) (Langmead and Salzberg, 2012) was used to identify any residual host genome in the metagenomic content (with parameter ‘-n 0.2′), in which Homo sapiens (human) genome assembly GRCh38 (hg38) was used as the reference genome. The quality of reads was then checked by FastQC (version 0.11.9) tool (Sadeghi et al., 2021). Microbial taxonomic composition and their relative abundance were profiled and quantified in the groups using MetaPhlAn (version 3.0.12) (Beghini et al., 2021) with default parameters. MetaPhlAn3 synthesizes data based on mapping the reads to a predefined data set consisting of 1.1 million specific marker genes. Finally, due to the batch effects of different groups, removeBatchEffect function was employed from the limma package (Ritchie et al., 2015). We extracted Gammaproteobacteria-related data from the result and compared it across HC, CD, and UC cohorts at different taxonomic levels. In particular, we focused on and characterized the Enterobacteriaceae community in the gut microbiome, since the pathobionts of this family are proposed the leading bacterial triggers in IBD.

### Functional profiling and Enterobacteriaceae-related biosynthetic pathways

Functional analysis and Enterobacteriaceae-related biosynthetic pathways profiling was carried out using HUMAnN3 (version 3.0.0) (Beghini et al., 2021), mapping to the UniRef90 database. Linear discriminant analysis effect size (LEFSe) (Segata et al., 2011) was used to identify differentially abundant bacteria species between the groups and linear discriminant analysis (LDA) score was obtained (bacterial species with LDA score >2.0 and P < 0.05 were considered to be significantly discriminant). STAMP was used to identify differentially metabolic pathways (Parks et al., 2014) and correlation coefficients were determined using Spearman’s rank correlation, analyses were undertaken using the psych R package.

### Statistical analysis

False discovery rates were estimated by the Storey method for adjusting multiple comparisons to compare differences between CD, UC, and HC cohorts. Other statistical analyses were conducted using R (version 3.5.2). For the significantly different species wad used the nonparametric factorial Kruskl-wallis test, *P* < 0.05 was considered a significant difference.

## Results

### Changes in the abundance of Enterobacteriaceae in patients with IBD

Analysis of the gut microbiome composition and richness indicated that the microbial community of both CD and UC cohorts was significantly enriched in Enterobacterales order compared to the HC cohort. Moreover, compared to the gut microbiome of HC subjects, CD patients exhibited a significantly elevated level of Enterobacteriaceae and Morganellaceae at the family level. Taxonomic perturbations in UC patients mirrored the elevated abundance of Enterobacteriaceae, Hafniaceae, Morganellaceae, and Pasteurellaceae families. To further delineate the potential role of members of the Enterobacteriaceae in the etiology of IBD, we conducted a pairwise comparison among the groups to investigate the changes in the relative abundance of genera and specie belonging to the Enterobacteriaceae in CD and UC groups relative to the HC cohort. Outstandingly, genera *Escherichia*, *Citrobacter*, *Klebsiella*, *Morganella*, and *Proteus* were significantly enriched in the gut bacterial community of both CD and UC patients compared to HC subjects.

**Figure 1:**
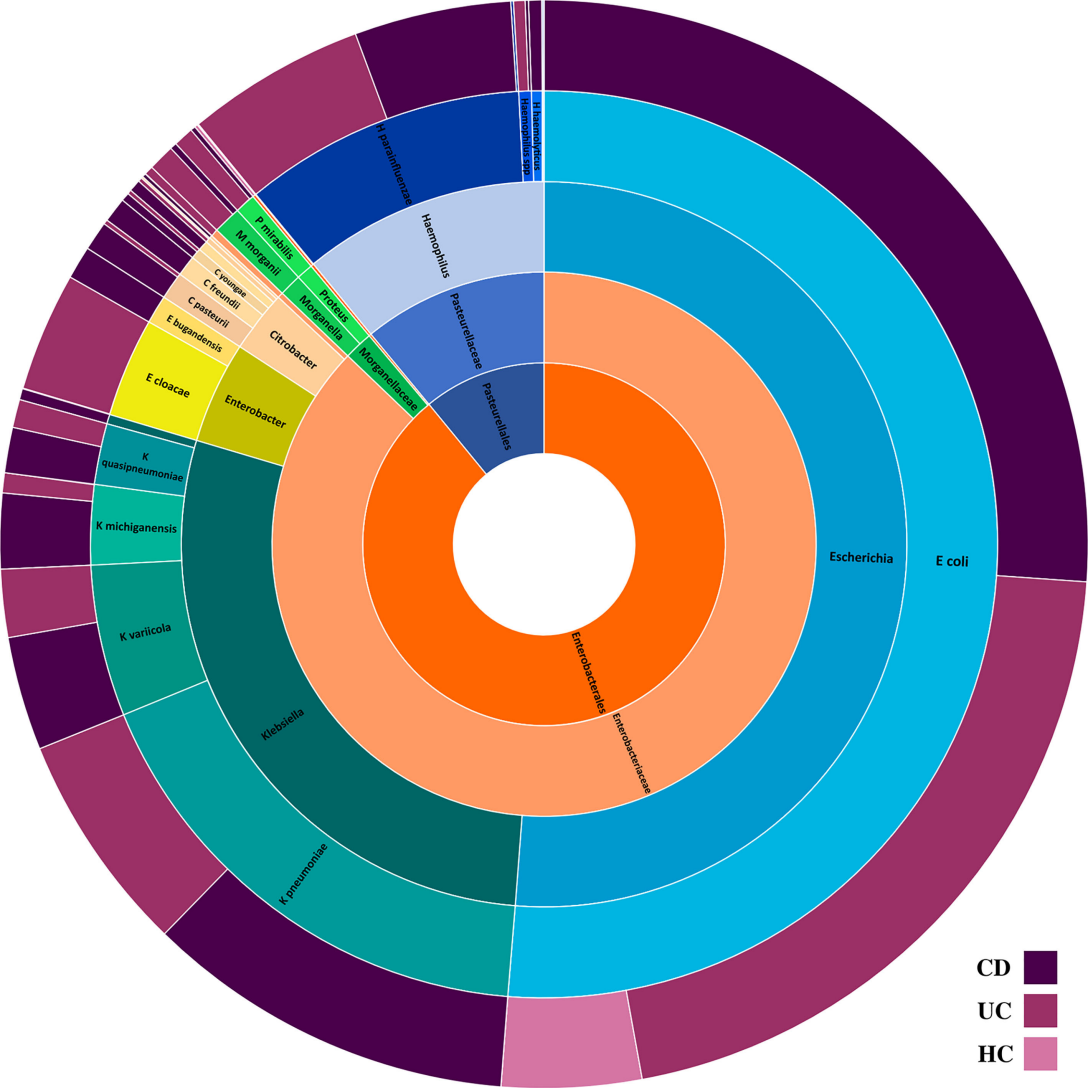
The composition of intestinal Proteobacteria in the CD, UC, and HC groups. Different colors represent different taxa, blue for *Escherichia* spp., teal for *Klebsiella* spp., yellowgreen for *Enterobacter* spp., peach for *Citrobacter* spp., green for *Morganella* and *Proteus* spp., light orange for Enterobacteriaceae family, dark orange for Enterobacteriales order, and steel blue for Pasteurellales order.

**Figure 2:**
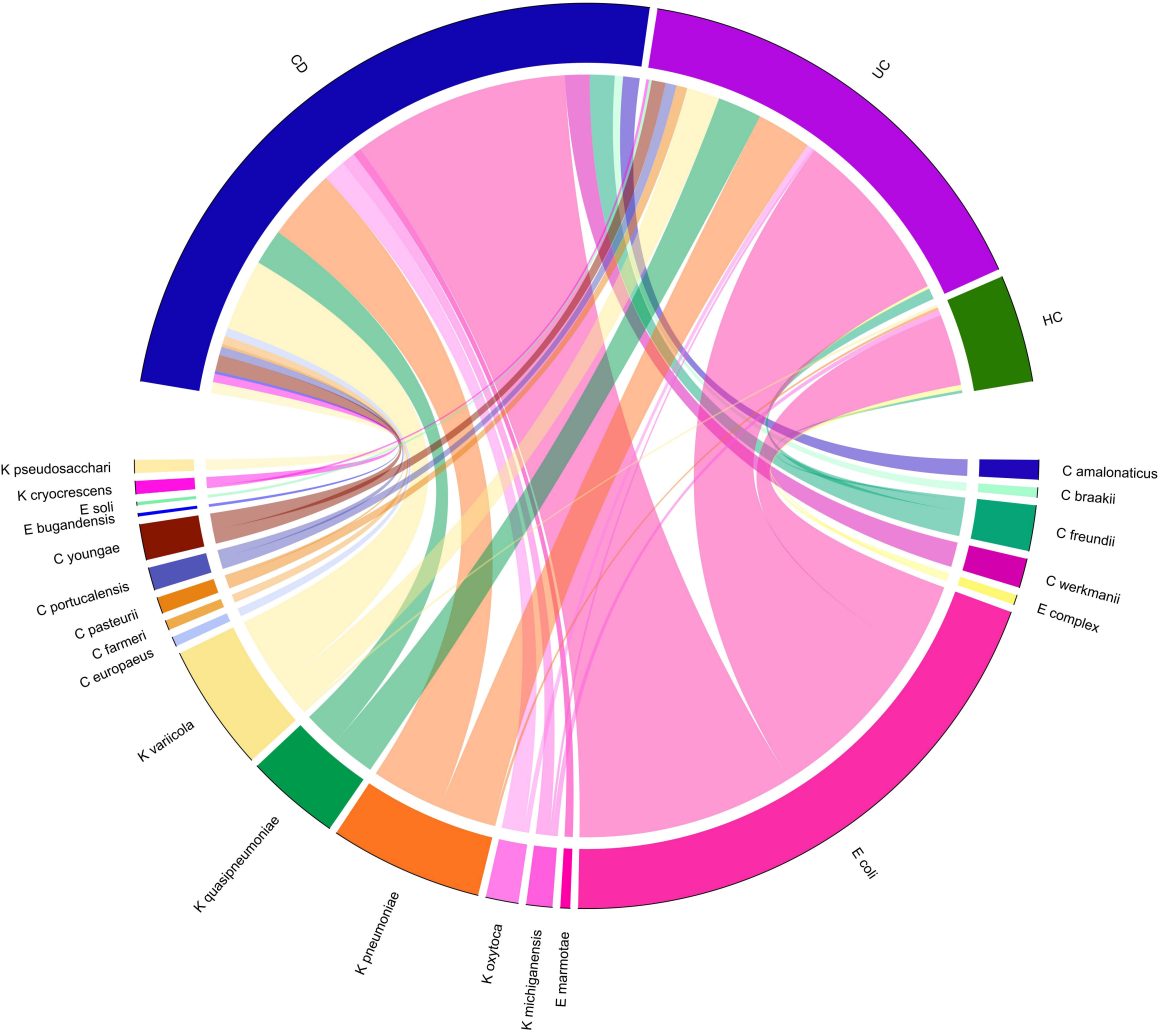
Chord-diagram visualizing the relative abundance of major Enterobacteriaceae species enriched in CD and UC compared to the HC groups. Circos plots show the correlation of the relative abundance of bacterial species within each group. Different Enterobacteriaceae species are separated by different colors.

A total of 14 Enterobacteriaceae species were differentially abundant in CD and/or UC patients, of which nine species were elevated in CD and 11 in UC patients relative to the HCs. Importantly, according to our metagenomics analysis, we did not find any bacterial species belonging to the Enterobacteriaceae that was significantly depleted in CD or UC patients relative to the HC cohort. Six Enterobacteriaceae species were significantly elevated in both CD and UC patients, including *E. coli*, *Klebsiella variicola*, *Klebsiella quasipneumoniae*, *Klebsiella pneumoniae*, *Proteus mirabilis*, *Citrobacter freundii*, and *Citrobacter youngae*, while *Klebsiella oxytoca*, *Morganella morganii*, and *Citrobacter amalonaticus* were uniquely differentially enriched in the CD cohort. Four species were uniquely abundant in the UC cohort, including *Citrobacter portucalensis*, *Citrobacter pasteurii*, *Citrobacter werkmanii*, and *Proteus hauseri*. The relative abundance of fecal Gammaproteobacteria in the CD, UC, and HC groups is illustrated in **Figure 1** according to the different taxonomic levels. **Figure 2** indicates a chord diagram visualizing the relative abundance of major Enterobacteriaceae species enriched in CD and UC compared to the HC groups. Our analysis also showed that 27% of the CD and 22% of the UC patients had a dramatically increased abundance of *E. coli* as over 10% of their intestinal Proteobacteria community, while only 2% of HCs had an abundance of *E. coli* as nearly 1% of their gut Gammaproteobacteria.

### Functional changes and stratified analyses of metabolic pathways by bacterial species

**Figure 3:**
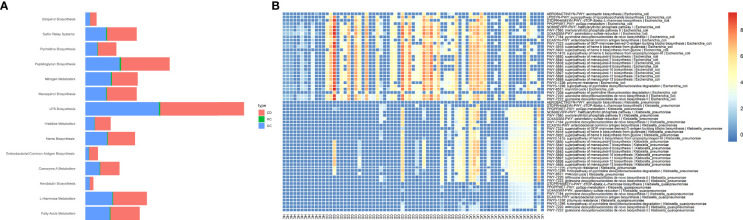
Potentially associations between metabolic pathways encoded by Enterobacteriaceae species and CD and UC as determined by Wilcoxon rank-sum test. To illustrate the enrichment of putatively species-specific pathways that were altered in CD and UC, we specifically focused on the subset of associations that were statistically significant (*P*<0.05), including *E. coli*, *K. pneumoniae*, and *K. quasipneumoniae*-related pathways. All associations were representative of positive correlations, *i.e.*, were elevated in both CD and UC patients compared to HCs. **(A)** The relative abundance of major microbial biosyntetic pathways that were involved in a positive association with the CD and UC cohorts. **(B)** The heat map represents metagenomically differences in the abundance of species-specific metabolic pathways in the intestinal bacteria community of CD, UC, and HC cohorts.

The predicted functional potential of the gut bacteria and their metabolic pathways in CD and UC samples was identified using HUMANn3. To investigate the bacterial species involved in metabolic pathways, we stratified analyses by bacterial species. We found 340 unique pathways among three groups, which included 3868 species-specific pathways and 21 different bacteria participating in these pathways. Enrichment analysis for altered metabolic pathways indicated that 270 unique bacterial pathways were specifically overrepresented in CD patients compared to the non-IBD controls (*P*-value < 0.05). Prominently, the relative abundance of *E. coli*-related metabolic pathways was significantly elevated in the CD group compared to the HC cohort, including 265 differentially abundant pathways. These findings indicated that *E. coli* participates in 94.8% of differential metabolic pathways between CD and healthy people. On the contrary, UC patients exhibited 59 metabolic pathways that uniquely passed the significant thresholds (*P*-value < 0.05) compared to the HC cohort, in which *K. pneumoniae-*related pathways accounted for 55/59 (93.2%). The differences in the relative abundance of the major species-specific metabolic pathways associated with CD and UC are shown in **Figure 3**. **Table 1** summarizes the major bacterial metabolic pathways and their associations with IBD according to the previous research.

**Table 1:** Major metabolic pathways used by Enterobacteriaceae species and their associations with CD and UC.

Pathway	Pathway class	Description	Evidence and impact mechanism implicated in IBD pathogenesis
AEROBACTINSYN-PWY	Siderophore and metallophore biosynthesis	Biosynthesis of aerobactin siderophore	- Aerobactin genes are present in a larger percentage of human enteric E. coli isolates of the B2 and D phylotypes (Nowrouzian et al., 2006; Dogan et al., 2014). - Non-catecholate siderophore systems are overrepresented in the *E. coli* population recovered from CD patients compared to healthy controls (Dogan et al., 2014). - Aerobactin could induce intestinal inflammation during colonization of mouse models (Torres et al., 2012). - Aerobactin enhances intracellular survival in macrophages and colonization of the mouse intestine by AIEC NRG 857c (Nash et al., 2010).
LPSSYN-PWY	Fatty acid and lipid biosynthesis	Biosynthesis of bacterial lipopolysaccharides (LPS)	- The patients with CD showed a statistically significant elevation of the lipid A antibody titers despite the absence of systemic endotoxemia (Kruis et al., 1984). -LPS can promote the development of bowel inflammation in the model of colitis by shaping the innate immunity *via* TLR4-dependent signaling mechanisms (Gronbach et al., 2014b). - The polarization of macrophages, mediated by gut-derived LPS, plays a key role in the regulation of inflammatory diseases (Wang et al., 2020).
ECASYN-PWY	Polysaccharide biosynthesis	Biosynthesis of the enterobacterial common antigen	- Enterobacterial common antigen-induced lymphocytes could promote enteric inflammation in IBD (Bull et al., 1971). - Enterobacterial common antigen interacts with the host immune system, leading to the production of broadly cross-reactive antibodies as well as proinflammatory cytokines (Rai and Mitchell, 2020).
PWY-5675	Nitrate reduction	Nitrate assimilation for synthesis of nitrogen-containing biomolecules	- Nitrogen metabolism pathways were significantly enriched in stool samples of pediatric CD patients (Lewis et al., 2015).
SO4ASSIM-PWY	Assimilatory sulfate reduction	Assimilatory sulfate reduction for the purpose of incorporation into newly synthesized molecules	- Sulfur relay systems were significantly enriched in stool samples of pediatric CD patients (Lewis et al., 2015).
PWY-5840	Menaquinol biosynthesis	Menaquinol-biosynthesis as a reversible redox component of the electron transfer chain and as a vitamin (vitamin K2)	- Menaquinone biosynthesis pathway abundance is significantly higher in the IBD cohort than in healthy adult volunteers (Chen et al., 2020).
PWY0-1298	Pyrimidine nucleotide degradation	pyrimidine deoxyribonucleosides degradation as total sources of carbon and energy	- Dihydroorotate dehydrogenase, an enzyme of the *de novo* pyrimidine biosynthetic pathway, strongly affects biofilm formation and promotes the ability of AIEC to grow in a macrophage (Rossi et al., 2022). - Intracellular pyrimidine concentrations act as a regulatory signal for genes involved in the virulence of AIEC strains (Rossi et al., 2022).
RIBOSYN2-PWY	Flavin biosynthesis	Riboflavin is the precursor for the essential flavin cofactors FMN and FAD, which are used in a wide variety of redox reactions.	- Riboflavin metabolism modules were overrepresented in ileal CD (Morgan et al., 2012). - Riboflavin metabolism is required for the microbiome’s response to the oxidative stress (high levels of reactive oxygen and nitrogen species) of the inflamed IBD gut (Morgan et al., 2012).

More important metabolic pathways encoded by the gut Proteobacteria that were at significantly higher levels in the CD samples than HC cohort included aerobactin biosynthesis (AEROBACTINSYN-PWY); superpathway of lipopolysaccharide [LPS] biosynthesis (LPSSYN-PWY); enterobacterial common antigen biosynthesis (ECASYN-PWY); superpathway of GDP-mannose-derived O-antigen building blocks biosynthesis (PWY-7323); ppGpp metabolism (PPGPPMET-PWY); nitrate reduction V (PWY-5675); SO4ASSIM-PWY (assimilatory sulfate reduction I); NONMEVIPP-PWY (methylerythritol phosphate pathway I); PWY-7560 (methylerythritol phosphate pathway II); DTDPRHAMSYN-PWY (dTDP-&beta;-L-rhamnose biosynthesis); PWY-7184 (pyrimidine deoxyribonucleotides *de novo* biosynthesis); IPWY0-166 (superpathway of pyrimidine deoxyribonucleotides *de novo* biosynthesis); FAO-PWY (fatty acid &beta;-oxidation I); ubiguinol biosynthetic pathways [including PWY-5855 [ubiquinol-7 biosynthesis (early decarboxylation)], PWY-6708 [ubiquinol-8 biosynthesis (early decarboxylation)], PWY-5856 [ubiquinol-9 biosynthesis (early decarboxylation)], PWY-5857 [ubiquinol-10 biosynthesis (early decarboxylation)], UBISYN-PWY: superpathway of ubiquinol-8 biosynthesis (early decarboxylation)]; and heme biosynthetic pathways [HEME-BIOSYNTHESIS-II (heme b biosynthesis I), HEMESYN2-PWY (heme b biosynthesis II (oxygen-independent)), PWY-5918 (superpathway of heme b biosynthesis from glutamate), PWY-5920 (superpathway of heme b biosynthesis from glycine), and PWY0-1415 (superpathway of heme b biosynthesis from uroporphyrinogen-III)]. Remarkably, *E. coli* was the only contributor to these metabolic pathways.

Analysis of species-specific metabolic pathways associated with UC indicated that the menaquinol biosynthetic pathways PWY-5850, PWY-5840, PWY-5838, PWY-5845, PWY-5896, PWY-5897, PWY-5898, PWY-5899 (superpathways of menaquinol -6, -7, -8, -9, -10, -11, -12, and -13 biosynthesis); PWY0-1338 (polymyxin resistance); PWY0-1586 (peptidoglycan maturation); RIBOSYN2-PWY (flavin biosynthesis I); PWY0-1298 (superpathway of pyrimidine deoxyribonucleosides degradation); NAGLIPASYN-PWY (lipid IVA biosynthesis); and PWY-6531 (mannitol cycle) were the most prominent metabolic signature of this cohort compared to the HC cohort, which belonged to *K. pneumoniae*. In particular, enrichment of *K. pneumoniae*-related metabolic pathways was strongly associated with UC patients. We also compared the differences in species-specific metabolic pathways between CD and UC patients. Our analysis revealed only two differentially overrepresented metabolic pathways between CD and UC groups, including PWY-7209 (superpathway of pyrimidine ribonucleosides degradation) and PWY-7409 (phospholipid remodeling), which significantly enriched in CD patients and belonged to *E. coli*.

## Discussion

Pathologic alterations in the gut microbial community and the host are crucial for activating a mucosal immune response, subsequently, initiating and perpetuating the chronic intestinal inflammation and thus being involved in the pathogenesis of diseases such as IBD. To date, experimental and computational evidence supports a sharp increase in the dominance of potentially harmful Gammaproteobacteria, especially species belonged the Enterobacteriaceae family, in patients with IBD (Morgan et al., 2012; Mukhopadhya et al., 2012a; Lavelle et al., 2015). Moreover, it has been realized that individual Gammaproteobacteria could be implicated as the trigger of the host’s inflammatory response, most likely caused by Enterobacteriaceae blooming, which leads to the development and course of IBD in genetically and/or immunologically predisposed hosts (Mukhopadhya et al., 2012a; Vester-Andersen et al., 2019). These studies have well documented the unique abilities of various members of the Gammaproteobacteria class that enable them to subvert and exploit host immune responses, and then, promote proinflammatory signaling and cytokine production in susceptible hosts (Sekido et al., 2020). Gut microbiota analyses of IBD patients have revealed disproportionate increases in mucosa-associated members of the families Enterobacteriaceae (Frank et al., 2011), Moraxellaceae (Wang et al., 2015), and Pseudomonadaceae (Wagner et al., 2008) in the Gammaproteobacteria class. In consonance with these reports, our metagenomics analysis revealed that the taxonomic and functional composition of intestinal bacteria in individuals with CD or UC was greatly shifted to Enterobacteriaceae and Morganellaceae-rich communities compared to the non-IBD participants, and the change in their relative abundance was generally confined to an overabundance of the genera *Escherichia*, *Klebsiella*, and *Citrobacter*. In the study conducted by Willing *et al.*, it was reported that individuals with predominantly ileal CD had a dramatically increased abundance of Enterobacteriaceae as high as 86% of the entire intestinal bacterial community (Willing et al., 2009).

According to our metagenomics analysis, *E. coli* in particular contributed to a large number of shifts in both CD and UC cohorts, in which the rate of shifts is approximately half that seen for the metagenomes. Previously, Martin et al. reported an increased mucosa-associated and intramucosal Gram-negative bacteria in colonic biopsy specimens of CD patients, of which 73% were identified as *E. coli* (Martin et al., 2004). So far, this facultative anaerobic bacterium has been studied extensively in patients with CD and is of particular interest owing to some of its strains’ behavior as “pathobionts” (Mirsepasi-Lauridsen et al., 2019). These pathobiont strains, designated adherent-invasive *E. coli* (AIEC), exhibit specific pathogenic properties, including the ability to adhere to intestinal epithelial cells (IECs); the capability to invade lamina propria and Peyer’s patches through M cells; the ability to survive within macrophage cells due to host autophagy defect; and finally, the ability to induce TNF-α from infected macrophages (Shawki and McCole, 2017; Nadalian et al., 2021). Evidence regarding AIEC strains in CD has taken decades to accumulate, with studies describing increased numbers of *E. coli* strains with specific virulence properties isolated from mucosal biopsy and resected surgical specimens of active CD patients (La Ferla et al., 2004; Meconi et al., 2007); *in vitro* observation of the same histological characteristics of AIEC strains, such as forming multinucleated giant cells along with the subsequent recruitment of lymphocytes (Meconi et al., 2007); and finally, the pathogenic distinction of strains isolated from CD patients and healthy individuals (Small et al., 2013; Palmela et al., 2018).

Despite several pieces of evidence that exist on the associations between AIEC and CD, the potential for these *E. coli* pathobionts to develop or facilitate UC is less documented mainly due to the lack of relevant animal models. So far, few studies have addressed the prevalence of AIEC isolates from the ileal and colonic biopsies of active UC patients, however, these reports suggest that UC‐associated *E. coli* are distinct from those associated with CD (Nadalian et al., 2021). On the other hand, some other pathotypes of *E.coli*, including diffusely adherent *E. coli* (DAEC) (Schultsz et al., 1997) and α-hemolysin-expressing strains have been previously linked to UC, as they were found to be able to strongly stimulate the secretion of the proinflammatory cytokines and disrupt tight junctions in Caco-2 cells, increasing barrier permeability (Jensen et al., 2015; Mirsepasi-Lauridsen et al., 2016).

The recognition of the overgrowth of specific *E. coli* strains as an important finding in observational research concerning IBD has been in place for nearly a decade; however, whether *E. coli* overgrowth is the trigger of the disease development or merely a subsequent effect of chronic intestinal inflammation remains unclear. Some evidence exists in the abnormal host response to IBD-associated *E. coli* pathobionts, suggesting drives the onset of IBD, which could support the ‘cause’ argument; however, other studies show clearly that inflammation in itself can result in the overgrowth of mucosa-associated *E. coli*, supporting the ‘effect’ argument (Mirsepasi-Lauridsen et al., 2019).

*Klebsiella* species, including *K. variicola*, *K. quasipneumoniae*, *K. pneumoniae*, and *K. oxytoca*, were also more abundant in CD and UC patients compared to the HC cohort. A considerable amount of *in vitro* and *in vivo* evidence exists suggesting that *K. pneumoniae* seems to have a key role in the initiation and perpetuation of the pathological damage involving the intestine and joint tissues in patients with IBD (Lee and Kim, 2011; Rashid et al., 2013). Furthermore, the ability of these pathobionts to outgrow other bacteria in the inflamed gut is supported by animal models and case studies, in which *Klebsiella* species and other related bacteria associated with the oral cavity are more abundant in the guts of IBD patients compared to healthy people (Carvalho et al., 2009; Atarashi et al., 2017). According to the study by Schirmer et al., oral pathobionts of *Klebsiella* are able to induce T helper 1 cells and elicit severe gut inflammation in genetically susceptible gnotobiotic mice (Schirmer et al., 2018). Moreover, multi-omics research on IBD has revealed that *Klebsiella* species are also more abundant in IBD cohorts (Lloyd-Price et al., 2019). The specific mechanisms involved in the translocation of oral *Klebsiella* pathobionts and inflammatory responses to intestinal *Klebsiella* species in IBD, however, remain to be elucidated.

Although taxonomic imbalances in the gut bacterial community of IBD patients have been long described, functional disruptions in gut microbial pathways have recently been reported to have a greater impact. Functional analysis of metabolic pathways encoded by the microbial community can provide important insights and opportunities for mechanistic studies on IBD, however, a majority of previous IBD research has described alternations in the composition of the gut microbiome, but the functional roles of the dysbiotic community remain less clear.

Metagenomic sequencing analysis has the potential to explore IBD-relevant microbial metabolic pathways by identifying metabolic alterations in the patients. In general, microbial metabolic pathway abundances are more consistently perturbed in IBD (Schirmer et al., 2018; Lloyd-Price et al., 2019). Moreover, a number of converging strands of metagenomics, metatranscriptomics, and metabolomics research into IBD have shown that the intestinal bacteria of IBD patients encode more oxidative stress (which is more favorable to Enterobacteriaceae) and nutrient transport pathways and fewer pathways related to amino acid synthesis and carbohydrate metabolism (Morgan et al., 2012; Gevers et al., 2014). Multi-omics data of stool samples from CD and UC patients showed that gut microbial metabolites such as bacteriocins, short-chain fatty acids, and nitrogen scavenging metabolites have displayed direct and indirect relationships to IBD pathogenesis (Schirmer et al., 2019). In this study, we investigated the difference in the functional composition of intestinal Gammaproteobacteria in CD, UC, and healthy people and found that many metabolic pathways enriched in the CD and UC groups. More importantly, our findings indicated that the changes in metabolic pathways were mainly related to *E. coli* in CD and *K. pneumoniae* in UC cohorts.

The top five most notable pathways, significantly elevated in the CD samples compared to the HCs, were biosynthetic pathways of aerobactin siderophore, LPS, enterobacterial common antigen, superpathway of GDP-mannose-derived O-antigen building blocks, and ppGpp encoded by *E. coli* strains. These inflammation-inducing factors are of particular importance for intestinal inflammation because of their ability to induce the secretion of large amounts of proinflammatory cytokines, such as TNF-α, IL-8, and IL-1β (Mirsepasi-Lauridsen et al., 2019). Previously, *E. coli* belonging to phylogroup B2 that overexpressed aerobactin-mediated iron uptake proteins were more frequently isolated from inflammatory and unchanged mucosa of active-phase IBD patients (Micenková et al., 2018). It has been also well documented that endotoxicity of LPS produced by intestinal Enterobacteriaceae plays a critical role in the induction of intestinal colitis by shaping the innate immunity *via* TLR4-dependent signaling mechanisms (Gronbach et al., 2014a). In addition, gut-derived LPS can promote macrophage accumulation and increase the proliferation of the macrophages *via* a CD14-dependent pathway (Wang et al., 2020). Therefore, the differences we found in the biosynthesis of gut-derived microbial factors that trigger host inflammation may correlate with host inflammation status in IBD patients and needs to be experimentally studied in the future. Other important pathways that were differentially abundant in CD patients were nitrogen metabolism, sulfur relay systems, and ubiguinol biosynthetic pathways belonging to *E. coli*. An analysis of fecal microbial gene pathways in pediatric CD patients using Random Forest revealed that nitrogen metabolism and sulfur relay systems were enriched in the CD fecal samples (Lewis et al., 2015).

Specifically, we show that various pathways involved in the menaquinol biosynthesis, which is responsible for vitamin K biosynthesis by intestinal commensal bacteria, are associated with UC patients and belonged to *K. pneumoniae* strains. Humans are unable to synthesize menaquinone and it is bacterially produced by intestinal commensal microorganisms which contribute to vitamin K requirements in humans. Enterobacteriaceae species are known to be important bacteria for the biosynthesis of menaquinone, a growth-promoting factor for a variety of microorganisms in the gut microbiota (Chen et al., 2020). In line with this, our finding of the elevated menaquinone biosynthesis pathway in UC patients could be attributed to the overgrowth of *K. pneumoniae*, which is a key IBD-associated pathobiont. However, in contrast to our finding, previous authors reported that a reduction in vitamin K synthesis by the gut microbiota may lead to a reduction in vitamin K levels in IBD patients (Walther et al., 2013).

The data presented here indicate that CD and UC in particular are characterized by an overrepresentation of Enterobacteriaceae bacteria mainly *E. coli* and *Klebsiella* species. These alternations in the intestinal bacterial community are also associated with major perturbations in the gut microbiome metabolic pathways, which revolved around the disproportionate presence of the biosynthetic pathways involved in virulence factors, oxidative stress, and perturbed nutrient availability during tissue damage. Taken together, our findings suggest that IBD-associated *E. coli* and *Klebsiella* species might play a role in the development and progression of IBD and may also play a role in disease relapses. Several genetic, immunological, and environmental factors (especially antibiotic therapy with broad-spectrum agents) could be associated with the overgrowth of Enterobacteriaceae in the GI microbiome, and hence, these must be taken into account during future studies on IBD. Further research, particularly including metabolomics, transcriptomics, and proteomics analysis along with dietary and antibiotic exposures, must be performed to additionally define the consequences of the overrepresentation and overactivation of Enterobacteriaceae species, particularly *E. coli*, in the gut of IBD patients and the specific mechanisms by which they are carried out or regulated by these bacteria.

## Data availability statement

The original contributions presented in the study are included in the article/supplementary material. Further inquiries can be directed to the corresponding author.

## Author contributions

BK, HH, and HAA designed the study. BahN and BanN collected data. BK performed the analyses. HH, BK, and EN-M interpreted the data. HH, BahN, and BanN wrote the article with input from all authors. HH, HAA, and EN-M intellectually and critically revised the manuscript. All authors contributed to the article and approved the submitted version.

## Funding

This project was financially supported by the Research Institute for Gastroenterology and Liver Diseases affiliated with Shahid Beheshti University of Medical Sciences, Tehran, Iran (No. IR.SBMU.RIGLD.REC.1399.055)

## Acknowledgments

The authors would like to thank the members of the Research Institute for Gastroenterology and Liver Diseases for their support.

## Conflict of interest

The authors declare that the research was conducted in the absence of any commercial or financial relationships that could be construed as a potential conflict of interest.

## Publisher’s note

All claims expressed in this article are solely those of the authors and do not necessarily represent those of their affiliated organizations, or those of the publisher, the editors and the reviewers. Any product that may be evaluated in this article, or claim that may be made by its manufacturer, is not guaranteed or endorsed by the publisher.
